# Quantifying the Influence of Lexical Surprisal on Acoustic Speech Encoding While Controlling for Within‐Speaker Variability

**DOI:** 10.1111/ejn.70569

**Published:** 2026-07-02

**Authors:** Shyanthony R. Synigal, Michael P. Broderick, Edmund C. Lalor

**Affiliations:** ^1^ Department of Neuroscience University of Rochester Rochester New York USA; ^2^ Del Monte Institute for Neuroscience University of Rochester Rochester New York USA; ^3^ School of Engineering, Trinity Centre for Bioengineering and Trinity College Institute of Neuroscience Trinity College Dublin Dublin Ireland; ^4^ Department of Biomedical Engineering University of Rochester Rochester New York USA; ^5^ Center for Visual Science University of Rochester Rochester New York USA

**Keywords:** EEG, speaker variability, speech, surprisal, vocoding

## Abstract

There is substantial support for the idea that the listening brain makes predictions about upcoming speech and that these predictions are integrated with sensory input to influence perception. For example, the early auditory encoding of words appears to vary based on how those words semantically relate to their preceding context, suggesting that top‐down information might feed back to affect acoustic speech processing. However, the way in which speakers enunciate words can vary based on how well those words fit with their preceding context. This presents a potential confound to the interpretation of top‐down prediction in the listener. In this study, we address this possibility by assessing the influence of probability‐based predictions (word surprisal) on electroencephalographic (EEG) indices of acoustic speech processing while controlling for variations in speaker dynamics. We analyzed EEG from 14 adults who undertook a perceptual pop‐out task in which prior information enhanced the comprehensibility of degraded speech while acoustic information was held constant. Behavioral results confirmed the manipulation's effectiveness and were mirrored in the neural indices of word surprisal processing. Importantly, a positive relationship between word surprisal and EEG tracking of word acoustics emerged for degraded speech when prior information rendered it intelligible, but was absent when it was unintelligible, despite identical acoustic input across conditions. The difference in neural effects between conditions also correlated with the corresponding difference in behavioral pop‐out. These findings support the claim that top‐down word predictability influences the acoustic encoding of natural speech, independent of variations in speaker enunciation.

## Graphical abstract

Linguistic context has been shown to influence the fidelity of acoustic speech tracking, but it remains unclear whether this reflects listener‐driven processing or differences in speaker enunciation. To address this, we used identical presentations of vocoded speech, differing only in the availability of prior information. We show that word probability modulates acoustic processing only when prior information renders speech intelligible, even after controlling for speaker enunciation.

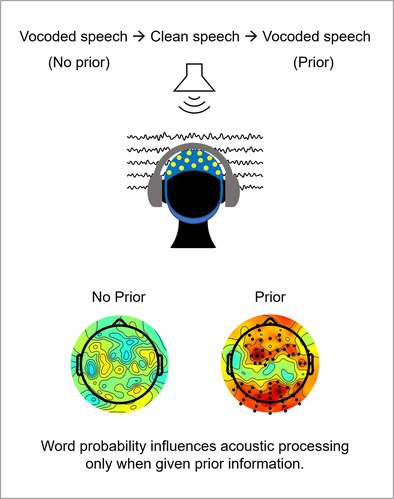

**Abbreviations**
ANOVA: analysis of variance
C: clean
CC: canonical components
EEG: electroencephalography
Hz: hertz
LME: linear mixed effects
MCCA: multiway canonical component analysis
ms: milliseconds
N400: negativity at 400 ms
NP: no prior knowledge
P: prior knowledge
PC: principal component
PCA: principal component analysis
REML: restricted maximum likelihood
SEM: standard error of the mean
TRF: temporal response function

## 1 Introduction

Human listeners have the ability to successfully perceive and comprehend speech, even in noisy environments. This ability is thought to be supported by integrating top‐down predictions based on prior knowledge with the speech input to inform perception (Baltzell et al. 2017; Di Liberto et al. 2018; Holdgraf et al. 2016; Sohoglu et al. 2012; Wild, Davis, and Johnsrude 2012). However, the precise mechanisms behind this integrative process are not fully understood.

One hypothesis is that sensory information and prior knowledge are integrated using only feedforward processes. In this account, predictions can be made but are utilized in a Bayesian fashion such that listeners are “ideal observers” who compare sensory input with stored representations and then select the most probable representation (Norris et al. 2016). An alternative hypothesis states that higher‐level information can modulate lower‐level processes using feedback connections. This hypothesis includes a range of theories from the TRACE model where feedback connections increase the activation of low‐level representations (McClelland and Elman 1986) to predictive coding where higher‐level representations predict lower‐level inputs. In predictive coding, the predictions or priors are compared with sensory input, and the difference (termed prediction error) is propagated up the system to update the predictions (Friston 2010; Lupyan and Clark 2015; Rao and Ballard 1999). Predictive speech processing has important clinical implications, as symptoms of schizophrenia (Corlett et al. 2019; Heinks‐Maldonado et al. 2007) and autism (Brock 2012; Pellicano and Burr 2012) are thought to result from an imbalance of top‐down and bottom‐up perceptual processes. Without a firm understanding of these processes, the field will remain limited in how well it can provide suitable interventions for those with atypical perceptual processing.

Improved understanding of the mechanisms underlying predictive speech perception would be helped by further characterizing neural responses to predictable speech. One particularly useful way to do this—from the perspective of understanding the phenomenon as it might operate in daily life—would be to do so in the context of continuous, naturalistic speech, where one could explore how natural variations in speech predictability affect the processing of speech acoustics.

Broderick and colleagues (Broderick et al. 2019; Broderick and Lalor 2020) introduced an analysis framework that could index the influence of higher‐level linguistic features on lower‐level acoustic‐phonetic processing. These studies involved analyzing EEG data collected from participants as they listened to continuous narrative speech. Specifically, the authors used a two‐stage regression analysis, where stage one involved modeling how the EEG reflected various low‐level acoustic‐phonetic speech features and stage two involved assessing how the fidelity of that encoding varied based on the semantic relationship between individual words and their preceding context (Broderick et al. 2019). In one of these studies, the authors explored how acoustic‐phonetic speech encoding was influenced by lexical surprisal (Broderick and Lalor 2020), which indexes how much information a new word adds to its preceding context (Kuperberg and Jaeger 2016). Words that are unexpected or less probable possess higher surprisal values and carry more information. Using this two‐stage regression analysis, the authors found that the more surprising a word, the better its acoustic and phonetic features were reflected in the EEG—possibly indexing the top‐down influence of prediction on bottom‐up acoustic‐phonetic speech processing (Broderick and Lalor 2020).

One potential issue with this approach, however, is that there is a well‐established relationship between the predictability of a word and how it is articulated by the speaker (Aylett and Turk 2004; Bell et al. 2009; Lieberman 1963). For instance, previous research has shown that speakers are efficient in delivering their speech (Jaeger 2010) and that speakers enunciate less predictable words “more carefully and more slowly” than words that are highly expected (Aylett and Turk 2004; Bell et al. 2009; Lieberman 1963). As such, it is possible that variations in the EEG tracking of acoustic‐phonetic speech features might be influenced by (or even solely reflect) differences in the speaker's articulatory dynamics, rather than by the listener's expectations. Although Broderick et al. (2019) tried to control for such systematic variations by including several additional regressors (e.g., envelope variability) in their analysis framework, it remains possible that speaker dynamics are playing a role in a measure that has been interpreted as a signature of predictive perception.

The goal of the present study was to definitively test whether speaker dynamics can fully explain the results of Broderick et al. (2019). We have done this using a paradigm that manipulated the comprehensibility of speech stimuli while controlling for speech acoustics. Specifically, we used a well‐known pop‐out task, where participants were presented with heavily degraded speech stimuli either with or without access to prior information (Di Liberto et al. 2018). Previous studies have shown that when such degraded speech is preceded by matching content, participants report an increase in speech clarity (Di Liberto et al. 2018; Sohoglu et al. 2012; Wild, Davis, and Johnsrude 2012), with several of these studies also showing related effects on neural indices of speech processing (Al‐Zubaidi et al. 2022; Sohoglu et al. 2012; Tuennerhoff and Noppeney 2016; Wild, Davis, and Johnsrude 2012). This pop‐out paradigm allowed listeners to hear the same stimuli in conditions where the speech could and could not be understood. As such, this paradigm allows us to address our research question because (1) the acoustics of the speech—and thus the speaker dynamics—do not change between two presentations of the same stimulus and (2) the intelligibility and predictability of the speech do change between two presentations of the same stimulus because prior information is available only for the second presentation. If the influence of context‐based predictions on speech acoustics is stronger for the condition in which the speech can be understood, it would represent strong support for the idea that listeners used context to predict the acoustic content of upcoming speech, beyond any effect that may derive from variations in the speaker's articulations.

## 2 Methods

### 2.1 Participants

Fourteen healthy adults (7 females, 21–31 years old) participated in this study. Each participant provided written informed consent and reported no history of hearing impairment or neurological disorders. This dataset has been published previously in a study that investigated the effect of prior knowledge on the cortical tracking of low‐level speech features (Di Liberto et al. 2018). All procedures in that study were approved by the Ethics Committee of the School of Psychology at Trinity College Dublin.

### 2.2 Stimuli and Experimental Procedure

The stimuli were excerpts from two audiobooks, *The Old Man and the Sea* by Ernest Hemingway and *The Tell‐Tale Heart* by Edgar Allan Poe, read by the same American male speaker. Each trial consisted of three 10‐s‐long speech segments. The first segment was degraded (no prior knowledge, NP), the second was clean (C), and the third was degraded (prior knowledge, P). The third segment is labeled P because it is preceded by the clean segment which supplies prior information about what will be heard in P. The first and third segments were degraded using three‐channel noise vocoding (Shannon et al. 1995). Gaussian noise and the clean speech stimuli were filtered into three logarithmically spaced frequency bands from 70 to 5000 Hz (70–494–1680–5000 Hz) using Greenwood's equation (Greenwood 1961). The envelope of each speech band was used to modulate the Gaussian noise in the same frequency band, which created highly unintelligible speech stimuli. Before the full experiment, participants were presented with about 10 min of vocoded speech to familiarize themselves with noise vocoding.

The original experiment (Di Liberto et al. 2018) consisted of 93 standard trials and 27 deviant trials. For each standard trial, all three segments were derived from the same audio clip (i.e., the C segment was the original clean speech from *The Tell‐Tale Heart*, and the NP and P segments were the noise‐vocoded versions of that clean speech). Meanwhile, in the deviant trials, a random (~5‐s‐long) chunk of NP and/or P was replaced with vocoded speech derived from *The Old Man and the Sea*. After C was presented, participants were asked if NP and C were the same (standard) or different (deviant). After P was presented, participants were asked if P and C were the same (standard) or different (deviant). Participants answered using a scale of 1–5 with the following confidence levels: definitely a deviant, probably a deviant, I do not know, probably a standard, and definitely a standard. The EEG analysis below focused only on standard trials; thus, EEG responses to deviant trials were not analyzed in the present study.

### 2.3 Data Acquisition and Preprocessing

EEG data were recorded from 128 scalp electrodes (plus two mastoid channels that were not analyzed in this work). The data were filtered online using a 0–134 Hz range and acquired at a 512 Hz sampling rate with the BioSemi Active Two system. Presentation software from Neurobehavioral Systems (http://www.neurobs.com) was used to present the stimuli to participants through Sennheiser HD650 headphones, at a sampling rate of 44.1 kHz.

The EEG data were high‐pass filtered at 1 Hz and low‐pass filtered at 8 Hz using a Chebyshev Type II filter with a 1 dB passband attenuation. The high‐pass filter had a 0.5 Hz cutoff frequency and 60 dB stopband attenuation, and the low‐pass filter had an 8.5 Hz cutoff frequency and 80 dB stopband attenuation. Noisy channels were identified using several metrics: mean standard deviation across channels, kurtosis (to detect unusually “peaky” activity), spectral estimates, and the probability distribution of channel activity. The thresholds for artifact rejection were set to 3, 10, 5, and 10 standard deviations from the mean of each measurement, respectively. The noisy channels were recalculated based on the surrounding channels using spherical spline interpolation (Delorme and Makeig 2004). There were six channels interpolated on average across participants, trials, and conditions. The data were then downsampled to 128 Hz and re‐referenced to the global average of all 128 scalp channels. A total of 120 10‐s speech segments were used in the original study for each condition. In the present study, we removed the deviant trials and focused our analyses on the 93 standard trials where all three segments matched one another.

Because each speech segment was only 10 s in duration—meaning the total amount of data per participant was limited—we applied multiway canonical component analysis (MCCA) to identify shared neural activity among participants and then used that shared information to denoise the EEG (de Cheveigne et al. 2019). For this process, principal component analysis (PCA) was first applied to spatially whiten the data (Comon and Jutten 2010). The data were then concatenated across participants, and PCA was applied to the grouped data before performing the MCCA decomposition. Canonical components (CCs) that were weakly shared across participants were discarded, and the remaining components were projected back into the EEG space to reconstruct the denoised signals. Following previous studies (Broderick et al. 2021; de Cheveigne et al. 2019), the data for each participant were first reduced to 40 principal components prior to MCCA, and the first 110 canonical components were retained for reconstruction. This procedure was applied separately to the EEG data for each condition (NP, C, and P).

### 2.4 Stimulus Characterization

Similar to Broderick and Lalor (2020), we extracted various acoustic and linguistic features from the speech stimuli. Quantitative representations of those features were used as inputs to a two‐stage regression analysis to determine how word probability (represented by lexical surprisal) affects lower‐level acoustic speech tracking. As mentioned below and detailed in the next section, the first stage involved relating the EEG data to an acoustic representation of speech, and the second stage involved quantifying how the strength of this EEG–acoustic relationship for each word varied as a function of that word's contextual predictability.

#### 2.4.1 Envelope

We indexed a simple acoustic representation of the speech stimuli in the form of the broadband speech envelope. This feature is a reasonable choice for the current study because it captures a fundamental low‐level acoustic property of the speech stimulus (viz., its instantaneous energy), because of its importance to speech intelligibility (Ahissar et al. 2001; Drullman et al. 1994; Shannon et al. 1995), and because it has consistently been shown to be reflected in human neural activity during various degraded speech paradigms (Baltzell et al. 2017; Corcoran et al. 2023; Di Liberto et al. 2018; Holdgraf et al. 2016; Millman et al. 2015; Peelle et al. 2013; Sohoglu and Davis 2020). Moreover, our design is a within‐participant design where we will be exploring the effects of prediction on envelope tracking for the same stimuli within the same participants across conditions of intelligibility/predictability. The speech envelopes were computed on the clean versions of each segment. It was first obtained by filtering the speech into three logarithmically spaced frequency bands between 70 and 5000 Hz according to Greenwood's equation. Then, the absolute value of the Hilbert transform was calculated for each frequency band, and this result was averaged across all three bands.

#### 2.4.2 Lexical Surprisal

The strength of top‐down word predictability was represented by lexical surprisal. This measure captured how (un)likely a word is to occur given some preceding context. Surprisal values were calculated for each word using the Transformer‐XL model. This model contains a recurrence mechanism that allows it to build and reuse memory from previous segments. It also preserves the temporal information of previous word embeddings. This model was chosen because it would allow us to predict the probability of an upcoming word using the context from all preceding words. We took the softmax of the values from the output layer of the model to estimate the probability of each upcoming word. Lastly, the surprisal value of each word in the audiobook segment was quantified as the negative log of the probability of that word's occurrence (Dai et al. 2019). Then, to assess how the EEG was affected by the surprisal of the words, we created a regressor that was composed of a train of impulses, with each impulse being located at the onset of each word and with a height that corresponded to the surprisal of that word.

Calculating next word probability for the P condition is complicated by the fact that the participants recently heard the same words in the C condition. Assuming that clarity of the P condition is increased (in comparison to NP), we repeated the text from the clips and allowed Transformer‐XL to compute next word probability based on this combined representation so that the first iteration of the clip would correspond to C and the second iteration would correspond to P. With this method, we assumed that context is established during the C segment, and as participants listen to P, they compare the incoming words to the preexisting context from C in addition to previous words in P.

#### 2.4.3 Nuisance Regressors

We also computed a number of other features from the speech that we included in our analysis as nuisance regressors: envelope variability, relative pitch, and harmonic resolvability, which were all derived from the clean audio clips. These variables were included in our second‐stage regression analysis to account for acoustic variations in the speaker's voice especially because they may also correlate with the envelope, surprisal, and/or one another. Envelope variability is represented as the standard deviation of the speech envelope for each word. “Clear” speech, for example, which has greater envelope modulations than informal, conversational speech (Krause and Braida 2004), has been shown to benefit speech perception in noisy environments (Bradlow et al. 2003) and evoke larger neural responses in comparison to conversational speech (Cunningham et al. 2002; Uchanski 2005).

Relative pitch is a pitch measure normalized to the speaker's vocal range (Tang et al. 2017). In fact, relative pitch was shown to uniquely predict delta‐phase EEG (Teoh et al. 2019). Praat software (Boersma and Weenink 2013) was used to calculate the absolute pitch of the stimuli, which was then z‐scored to result in relative pitch. In addition, a previous fMRI study found regions in the auditory cortex that respond primarily to resolved harmonics (Norman‐Haignere et al. 2013). Resolvability is when the harmonics of a sound are processed within separate filters in the cochlea, whereas unresolved harmonics are processed within the same filter. Resolvability was calculated using custom MATLAB scripts based on a model of the human auditory periphery (McDermott and Simoncelli 2011; Teoh et al. 2019). Again, these three nuisance regressors were included to soak up variability in the EEG–acoustic relationship that might derive from how the speaker was producing the speech, allowing us to more cleanly assess how top‐down predictions in the listener were influencing that relationship.

### 2.5 Modeling the Relationship Between Speech Features and EEG Responses

**FIGURE 1:**
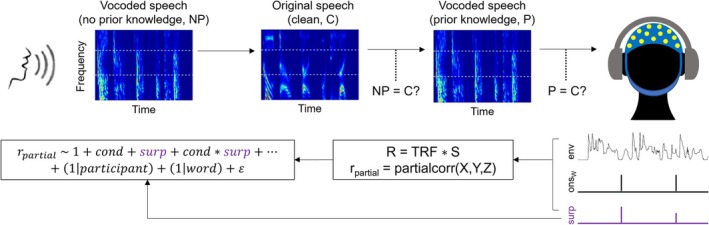
Experimental and data analysis methods. (Top) EEG data were recorded while participants listened to three 10‐s segments of speech (adapted from Di Liberto et al. 2018). The first (no prior knowledge, NP) and third (prior knowledge, P) segments were degraded using three‐channel noise vocoding and the second segment was clean (C). Only standard trials were used, where the speech in each segment was identical (albeit degraded in the NP and P conditions). After listening to C, participants were asked if it matched NP, and after listening to P, participants were asked if it matched C. (Bottom) A forward model (TRF) was used to predict the EEG (response, R) from three speech features (S): surprisal values (surp), word onset (ons_W_), and the speech envelope (env). Partial correlation coefficients were calculated for each model (r_partial_) using the actual EEG (X), the predicted EEG from one model (Y), and a concatenation of the predicted EEG from all other models (Z). A linear mixed effects (LME) model was then used to assess the relationship between surprisal and the envelope model partial correlation coefficients at the individual word level.

To relate the EEG to the acoustic and linguistic features of speech, we used forward encoding models. Forward models map from the stimulus feature of choice to the EEG responses. This method allows us to model discrete speech features (e.g., surprisal) and determine how the dynamics of those features influence the neural activity on each electrode. We computed forward models, specifically temporal response functions (TRFs), to capture how the EEG data varied as a function of two specific speech features, namely, the continuous acoustic envelope and lexical surprisal (Figure 1). As we will discuss in more detail below, our primary analysis centered on exploring how the acoustic envelope was tracked by the EEG and how this tracking varied as a function of lexical surprisal. However, we also used forward modeling to examine how lexical surprisal itself was tracked by the EEG as a means to determine whether or not participants were processing the linguistic information in the speech (Broderick et al. 2018). This was especially important to know in the context of the vocoded conditions.

Prior to modeling, each speech feature was normalized between 0 and 1, and the EEG responses were z‐scored. A nested 10‐fold cross‐validation procedure was used to fit the TRF models for each participant, where TRFs were trained on all but one fold and then tested by predicting the left out neural data. The neural response, *r(t,n)*, sampled at times *t = 1 … T*, with *n* electrodes, is the result of a channel‐specific TRF, *w(τ,n)*, convolved with a lagged stimulus feature, *s(t − τ)*. The residual response not explained by the model, *ε(t,n)*, is included in this expression as well. This stimulus–response mapping is represented as (Crosse et al. 2016)

$$r\left(t,n\right)=\sum_{\tau }w\;\left(\tau ,n\right)\;s\left(t-\tau \right)+\varepsilon \left(t,n\right)$$

For the lexical surprisal models, we used lags of −100–700 ms to capture longer latency responses reflective of context‐based word processing (Broderick et al. 2018). In addition, we trained envelope‐based forward models for use in the second‐stage regression, which included lags of −100–300 ms. In this case, the range was shortened to explicitly focus only on early acoustic processing in line with previous research. Model performance was assessed by computing the correlation between the actual EEG of the left‐out fold with the predicted EEG using Pearson's correlation coefficient. To control for overfitting, a regularization parameter was included when fitting the TRF models (Crosse et al. 2016, 2021). This parameter was chosen (during the nested cross‐validation process) as the value that produced the highest prediction accuracy most often across folds for the C condition. We then used this fixed regularization parameter value when training and testing models for both the NP and P conditions to allow a direct comparison across those conditions.

One challenge when modeling EEG responses to specific speech and language features is that many of those features are correlated with one another across time. For example, features such as word onsets are likely to correlate with our envelope and word surprisal predictors. As such, not accounting for the influence of word onsets on the EEG could confound the interpretation and performance of our forward models. To account for this possibility, we computed partial correlation coefficients (Pearson's *r*) to find the unique contribution of either the envelope or lexical surprisal to the EEG. Specifically, using the abovementioned time lags, we computed three sets of forward models: one based on the speech envelope, one based on lexical surprisal, and one based on word onsets. The word onset feature consisted of an impulse vector with a height equal to one at the onset of each word in the trial. Then, we used MATLAB's partialcorr (X,Y,Z) function (Fisher 1924) to calculate the partial correlation between the actual EEG (X) and the predicted EEG from either the envelope model or the surprisal model (Y) while controlling for the EEG activity that can be accounted for when models were trained on the other two features (Z, concatenation of the predicted EEG from the word onset and either the lexical surprisal or envelope models). This calculation was performed for each fold.

### 2.6 Assessing the Influence of Context‐Based Predictions on Acoustic Encoding

We wished to compute the influence of lexical surprisal on the acoustic encoding of each word in the P and NP conditions using a two‐stage regression analysis. As discussed in the previous section, the first stage involved calculating a forward encoding model based on the acoustic speech envelope, with lags from −100–300 ms and a partial correlation approach that controlled for the effects of word surprisal and word onset on the EEG. Correlations between the predicted EEG and the recorded EEG were calculated with Spearman's rho due to the short length of the words (compared to the length of the entire envelope where Pearson's *r* was used). Again, partial correlations and early lags were used to control for the effect of lexical surprisal and word onset and to focus exclusively on early acoustic processing.

Stage two of the regression aimed to assess how variations in the lexical surprisal of a word might influence that word's envelope encoding. To do this, we used a linear mixed effects (LME) model, which considered variations explained by multiple independent variables and additional variations not explained by the independent variables (Bates et al. 2014). The LME model was calculated in R (lmerTest Version 3.1‐3 and lme4 Version 1.1‐30 in R) with the default parameters that included fitting the models with restricted maximum likelihood (REML) and using Satterthwaite's method for the *t*‐tests. We utilized the following equation:

$$\begin{array}{c} r_{\text{partial}}\;∼\;1+\;\text{cond}\;+\;\text{surp}\;+\;\text{envStd}\;+\;f_{\text{rel}}\;+\;\text{res}\;\\ \;+\;\text{cond}\;*\;\text{surp}\;+\;\text{surp}\;*\;\text{envStd}\;+\;f_{\text{rel}}\;*\;\text{res}\;\\ \;+\;(1|\text{participant})\;+\;(1|\text{word})\;+\;\varepsilon \end{array}$$

The dependent variable is the envelope model partial correlation coefficient around individual words (Spearman's rho, *r_partial_*), and the independent variables are lexical surprisal (*surp*), stimulus condition (*cond*), and the nuisance regressors (envelope variability [*envStd*], relative pitch [*f*_*rel*_], and resolvability [*res*]). A dummy coding scheme was used for the condition variable where P = 0 and NP = 1. Because this equation includes all words in the stimuli and data from all participants, the model accounts for random effects caused by each word and participant, in addition to any other random error (Figure 1). Relative pitch and resolvability are two pitch‐related measures that have both been shown to be tracked by delta band EEG (Teoh et al. 2019), so the two were included in an interaction term. The interaction between surprisal and envelope variability was also included, given the similarities between the speech envelope and envelope variability and because the influence of surprisal on envelope encoding might be bigger for words that have larger envelope modulations. Because the nuisance regressors are the same for NP and P, the condition variable was not included in those interactions. There were 2961 words total with an average length of 244.7 ms and a standard deviation of 156 ms.

Like the Broderick 2019 study, we were mainly interested in early effects of top‐down processing, so we calculated partial correlation coefficients within the first 100 ms of each word's onset (Broderick et al. 2019). This window avoids longer lags where linguistic processing and integration occurs (Friederici 2011; Pickering and Gambi 2018). Therefore, finding effects of surprisal on speech envelope processing at such short latencies would provide strong evidence that listeners used this linguistic context to predict upcoming acoustic information. However, one could argue that surprisal might affect the dynamics of most of (if not the entire) word, so we also recalculated the LME models in a later window (100–200 ms following word onset) for completeness. Exploring effects at lags longer than 200 ms would surely be confounded by responses that reflect linguistic processing. The nuisance regressors were calculated across the same two windows of time. Each numeric independent variable was z‐scored prior to modeling.

### 2.7 Statistical Analysis

**FIGURE 2:**
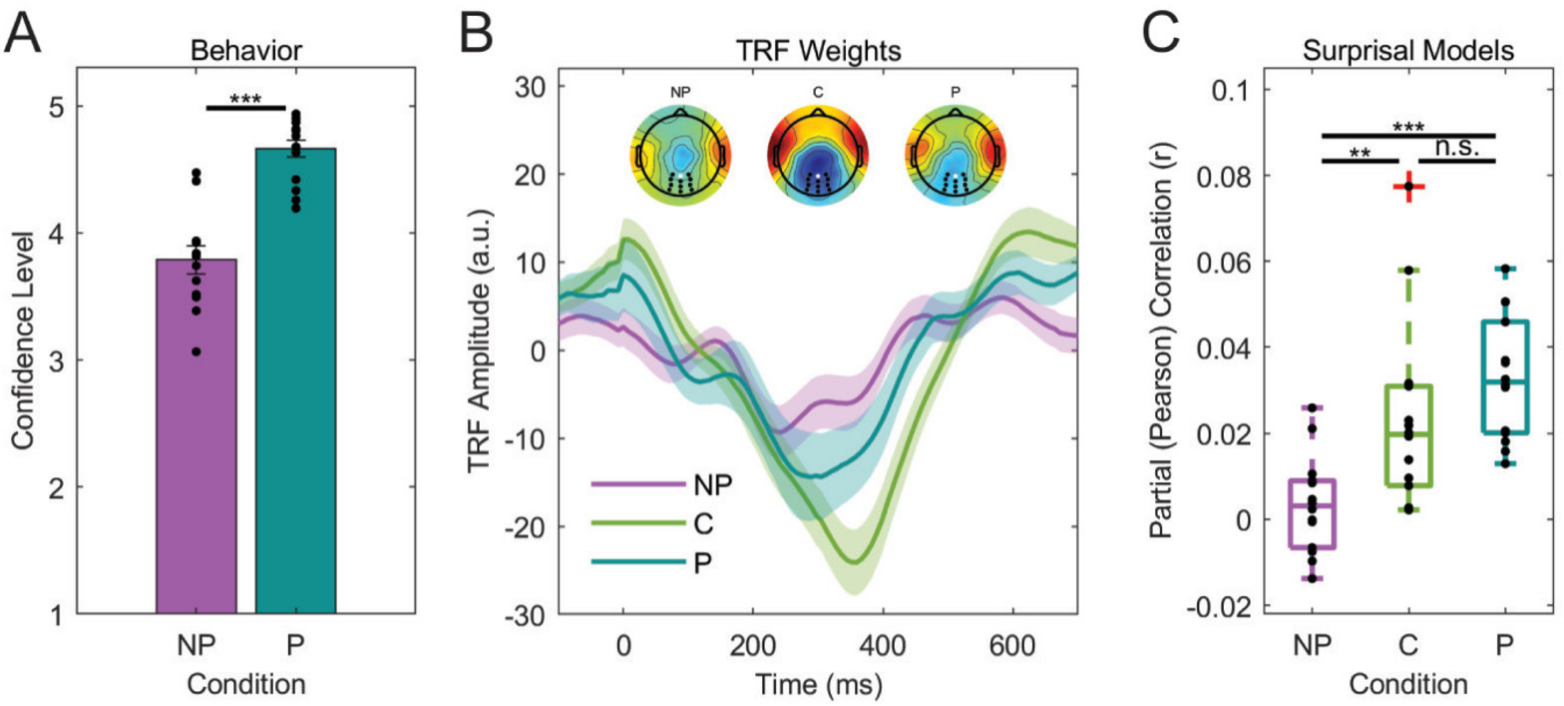
Behavioral and forward modeling results. (A) The average confidence level (+/−SEM) across trials and participants. Participants reported how sure they were that the degraded speech matched the clean speech on a scale from 1 to 5—essentially, 1, meaning they are definitely different, and 5, meaning they are definitely the same. Significance was determined using a Wilcoxon signed‐rank test. Each black marker represents a participant. (B) NP, C, and P surprisal TRF weights (+/−SEM) across participants. The topographies show N400‐like TRF weights for each condition at ~360 ms, where C peaks. Data from the electrode indicated by the white marker are plotted over time. (C) Surprisal model partial correlation coefficients for the NP, C, and P conditions. These were averaged across the 12 black and white marked channels from (B). Two‐tailed *t*‐tests followed by Bonferroni correction were used to determine the difference between conditions.

Statistical analyses were performed in R (Version 4.2.0; R Core Team 2022) and in MATLAB (MathWorks 2021). Wilcoxon signed‐rank tests were used to test the difference between the behavioral responses. Friedman test (non‐parametric version of repeated measures ANOVA) and pairwise Wilcoxon signed‐rank tests with Bonferroni corrections were used to determine differences in EEG partial correlations. Pairwise *t*‐tests were used to determine differences in EEG partial correlations from the envelope models, as we were only interested in differences between the degraded conditions in that analysis. Permutation testing was also performed to test if the partial correlation coefficients were greater than chance. After training and testing the TRF models, partial correlation coefficients were computed between the actual EEG (in its correct order) and shuffled predicted EEG trials. This was repeated 1000 times for each participant. For the analysis that focused solely on lexical surprisal encoding, the null partial correlations were averaged across folds, electrodes (12 parietal electrodes in Figure 2), and participants, and we assessed the proportion of coefficients that were greater than the actual group‐averaged coefficients. A similar procedure was conducted for the envelope models, except the null coefficients were averaged across 12 frontotemporal electrodes reflective of acoustic processing (Di Liberto et al. 2015).

Permutation testing was also conducted to test the significance of the LME model coefficients at each scalp electrode. To test this, the surprisal values were shuffled between the words 30 times (within each trial), and an LME model was calculated for each shuffle and participant. (To be clear, the surprisal impulses still occurred at word onset; only their values were shuffled between words.) The 30 models were averaged to result in a null model for each participant. Cluster‐based permutation tests were then performed between the actual and null models to find the electrodes where the model coefficients were significantly greater than chance. Additional computations were conducted to test if the surprisal coefficients were greater in the P condition compared to the NP condition. The cluster‐based permutations allowed for the correction of multiple comparisons at the electrode level such that samples with a *t*‐value greater than a threshold of 0.05 were selected and clustered according to other neighboring channels. Cluster‐level statistics were then calculated by summing the *t*‐values within each cluster, and the largest sum was used as the test statistic. Permutation tests with 1000 random permutations were performed, and the Monte Carlo method was used to approximate the *p*‐values (Maris and Oostenveld 2007).

## 3 Results

### 3.1 Prior Information Improves the Perception of Degraded Speech and the Cortical Tracking of Lexical Surprisal

We first wished to establish that the perceptual pop‐out task produced improvements in speech perception and the associated neural indices of linguistic processing. This was central to being able to claim that top‐down predictions influence acoustic speech processing independent of variation in the acoustics of the speech (because the NP and P acoustics are identical). As previously reported for this dataset (Di Liberto et al. 2018), prior information led to clearer perception of the noise‐vocoded speech, with participants being more confident that the P and C segments matched compared to NP and C (*p* = 6.104 × 10^−5^, one‐tailed Wilcoxon signed‐rank test; Figure 2A).

We expected this increase in speech perception to be accompanied by stronger neural indices of lexical surprisal processing for the P condition relative to the NP condition. The prior information from C should increase the clarity of words in the P segments, in turn giving participants better access to the words and allowing them to take advantage of word context in those clips. This should be reflected by larger TRF weights (reminiscent of the N400 ERP component [Broderick et al. 2018; Kutas and Hillyard 1980]) based on lexical surprisal, as well as greater EEG predictions (assessed via partial correlations) based on that TRF.

Here, forward modeling was used to predict each participant's EEG from the lexical surprisal values. Each word was evaluated on how surprising (or improbable) it was given the preceding words in the same segment. As described in Section 2, the C condition was included when calculating surprisal for P, as information in C helped improve the perceived clarity of P (Di Liberto et al. 2018). The P and NP/C surprisal estimates were significantly correlated (rho = 0.576, *p* < 0.001). Partial correlations were computed between the actual EEG and predicted EEG from the surprisal models while controlling for effects of word onsets and the speech envelope. Word onsets and the speech envelope were used here because these acoustic‐related features may independently influence EEG responses to the degraded speech segments. The TRF weights were averaged across the 14 participants with electrode Pz plotted over time (Figure 2B). The partial correlations were averaged across 12 parieto‐occipital electrodes for each participant (Figure 2C).

EEG partial correlations based on the lexical surprisal models were significantly greater than chance for the C (*p* < 0.001) and P (*p* < 0.001) conditions, but not for the NP condition (*p* = 0.666). The Friedman test reported a significant effect of condition (*Χ* ^2^(2) = 19, *p* = 7.485 × 10^−5^). The effect size for the difference between the three conditions was 0.68, which is considered a large effect (Kendall's W, nonparametric effect size calculation). The C and P condition partial correlation coefficients were significantly different from the NP condition values (C vs. NP, *p* = 0.005; P vs. NP, *p* = 3.662 × 10^−4^, paired two‐tailed Wilcoxon signed‐rank tests with Bonferroni correction). There was no difference in the partial correlation coefficients between C and P (*p* = 0.106). We also reran the P condition forward analysis using the same surprisal values as NP to ensure that the observed differences were not due to small differences in surprisal values. This repeated analysis once again showed that lexical surprisal independently predicted EEG to a greater degree in P compared to NP (*p* = 0.009, paired two‐tailed Wilcoxon signed‐rank test). Altogether, comparison between the P and NP results suggests that the improved perception of degraded speech based on prior knowledge was accompanied by enhanced neural responses to lexical surprisal.

### 3.2 EEG Signatures of Acoustic Speech Processing Are Stronger for Less Predictable Words, Beyond Variations in Speaker Articulation

Having established that the pop‐out paradigm led to improved speech perception in the P condition relative to the NP condition, we then wanted to assess whether context‐based prediction (i.e., lexical surprisal) had any influence on the encoding of the speech acoustics (as indexed by the EEG predictions based on the speech envelope). We expected this influence to be stronger for the P condition compared to the NP condition, which would indicate a role for lexical predictions independent of speech acoustics since the acoustic information in both conditions is the same. The first step of this analysis was to predict neural responses from the speech envelope using a forward model, followed by computing partial correlation coefficients. The next step was to perform the second‐stage regression analysis; for this, we computed envelope variability, relative pitch, and resolvability (the nuisance regressors). Then, an LME model was fit using single word envelope partial correlation coefficients as the dependent variable, lexical surprisal and nuisance regressors as fixed effects, and words and participants as random effects. Because EEG partial correlation coefficients are the dependent variable, a beta coefficient (or slope) was calculated at each electrode for each independent variable.

**FIGURE 3:**
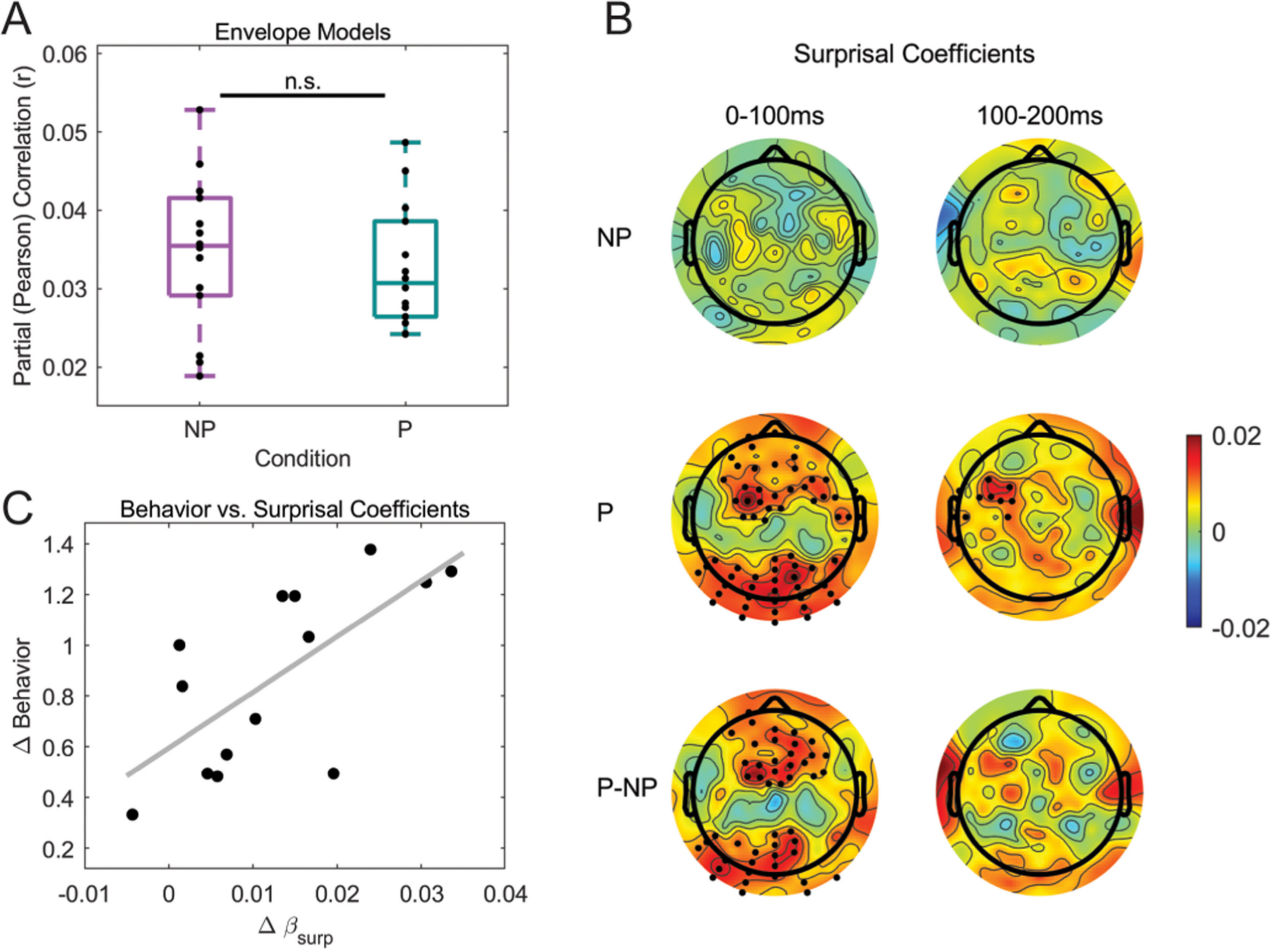
Two‐stage regression analysis. (A) Speech envelope model partial correlation coefficients for the NP and P conditions. Each marker represents a participant. Paired *t*‐tests were conducted to test the difference between the two conditions. (B) LME model surprisal coefficients in the first 0–100 ms and 100–200 ms following word onset for the NP and P conditions and the difference between the two. The black markers in the NP and P rows indicate electrodes that were significantly greater than chance as indicated by cluster‐based permutation tests. The black markers in the P–NP row indicate electrodes that had significantly larger surprisal coefficients in P compared to NP (also using cluster‐based permutation tests). (C) Pearson correlation of the change (P–NP) in behavior with the change in the surprisal coefficients. The plot displays a gray least squares line and a marker for each participant.

With regard to stage one of the two‐stage regression analysis, the speech envelope model partial correlation coefficients were greater than chance for both the NP and P conditions (*p* < 0.001, *p* < 0.001), highlighting that EEG robustly tracks the low‐level acoustics of noise‐vocoded speech irrespective of its intelligibility (MacIntyre et al. 2024). Furthermore, in spite of the differences in perceived clarity between the NP and P conditions, there was no difference in envelope model partial correlation coefficients between the two conditions (*t*(13) = 0.564, *p* = 0.583, paired two‐tailed *t*‐test; Figure 3A). This was not hugely surprising given the relatively large contribution of general auditory processing to EEG envelope tracking (Prinsloo and Lalor 2022).

We then examined the effects of surprisal on early single‐word encoding, indexed by envelope tracking accuracy during the 0–100 and 100–200 ms intervals following word onset, using the LME analysis described above (i.e., stage two of the regression analysis). The NP and P surprisal coefficients that resulted from the LME models are shown in the first two rows of Figure 3B. We sought to validate the coefficient *p*‐values using permutation tests, as the *p*‐values could be artifacts resulting from our large sample size (Lin et al. 2013). Cluster‐based permutations were conducted to test the significance of the model coefficients and to correct for multiple comparisons across neighboring electrodes. The permutation tests revealed surprisal coefficients that were greater than chance in the P condition (0–100 ms, two clusters, *p* = 0.006 and *p* = 0.008; 100–200 ms, one cluster, *p* = 0.038), but not the NP condition (*p* > 0.05 for both time ranges), indicating that surprisal influenced envelope tracking for the P condition, but not the NP condition. The LME models identified significant results for the other fixed effects as well, but only the resolvability coefficients in the 0–100 ms range were able to survive the permutation tests (Figure S1).

Cluster‐based permutation tests were also used to determine if the effect of surprisal on envelope tracking—as indexed by the surprisal coefficients of the LME model—were greater in the P condition compared to the NP condition for both time ranges. Here, the test revealed greater P condition surprisal coefficients at frontal and parieto‐occipital electrode locations in the 0–100 ms time window only (two clusters, *p* = 0.012 and *p* = 0.020). Like the TRF analysis from the previous section, we repeated the two‐stage regression using the NP surprisal values in the P condition to ensure the differences we saw did not solely result from the iteration method used when calculating the original P surprisal values. This new analysis showed similar results for the NP condition and also revealed P condition surprisal coefficients that were greater than chance in both time windows (0–100 ms, two clusters, *p* = 0.020 and *p* = 0.031; 100–200 ms, *p* = 9.990 × 10^−4^; Figure S2). Altogether, these results suggest that the more surprising a word, the stronger its acoustics are represented in EEG when the speech content can be perceived (i.e., in the P condition). Importantly, the acoustic properties of NP and P are the same, yet the LME analysis found a significant difference in surprisal coefficients between the two conditions. This suggests that the relationship between word probability and EEG partial correlations (from the envelope TRF model) is not fully explained by within‐speaker dynamics or the additional acoustic features included here.

Lastly, we examined if the influence of word surprisal on envelope tracking related to the participants' behavioral (confidence) scores. For this analysis, we computed the correlation between the change in the LME model surprisal coefficients (at the 48 significant electrodes resulting from the cluster‐based permutation tests) and the change in behavioral responses between the P and NP conditions using Pearson's correlation. We hypothesized that the change in the surprisal coefficients would be positively correlated with the change in behavior; since prior knowledge is known to improve speech clarity, it should then increase a participant's ability to use lexical predictions when processing speech acoustics. As expected, we found that the change in participants' behavioral response was significantly correlated with the change in their surprisal coefficients (*r* = 0.690, *p* = 0.003) within 100 ms after a word's utterance (Figure 3C). In summary, our results suggest that when prior information enabled the comprehension of degraded speech, word predictability influenced the envelope tracking of that speech.

## 4 Discussion

This study investigated the effect of word probability on acoustic tracking of degraded speech while attempting to control for systematic variations in speech acoustics that might arise from a speaker's articulations. Specifically, we analyzed data from a previously published study (Di Liberto et al. 2018), in which three‐channel noise vocoded speech was (P) or was not (NP) preceded by prior information (clean speech, C). Behavioral scores demonstrated that participants could better perceive the same degraded speech when it was preceded by prior information than when it was not. Forward models showed that this behavioral improvement was accompanied by increased EEG responses to contextual linguistic information carried by the words, as indexed by lexical surprisal. Central to our study, we found that the top‐down influence of word predictability on the encoding of individual word acoustics increased when degraded speech was preceded by prior information and was thus easier to understand. This increase in the influence of word predictability also correlated with the difference in participants' speech clarity ratings between conditions. This demonstrates that top‐down linguistic predictions influence bottom‐up acoustic processing in a way that cannot be fully explained by variability in the speaker's articulatory dynamics.

Our use of the vocoded pop‐out paradigm had the advantage that the talker's speed and articulations were identical across the NP and P segments. Plus, we took the additional step of controlling for various speech features that could explain speaker dynamics (e.g., envelope variability, resolvability, and relative pitch) in our second‐stage regression. The inclusion of these factors should reduce the effects of variation in speaker articulations that correlate with word surprisal—as might be expected from previous literature (Aylett and Turk 2004; Bell et al. 2009). Indeed, this was supported by the fact that we found no significant effect of word predictability on envelope tracking in the NP condition (Figure 3B). Meanwhile, the significant influence of surprisal on envelope tracking in the P condition (Figure 3B), even with these control factors included, suggests that word predictability has a significant effect on acoustic processing in listeners.

One limitation of this study was how the participants and/or our language models might be assessing lexical surprisal during this paradigm. For example, the audio clips in this paradigm were segmented from audiobooks. Since the clips were only 10 s long, a clip might start in the middle of a sentence or end in a fragment. So, the way Transformer‐XL calculated word probability or the manner in which participants encoded word surprisal may have differed had they been given all complete sentences from a continuous narrative. Future work should aim to take these into consideration when designing an experiment to assess context‐based features of speech processing. In any case—while it might be imperfect—it does not fatally confound our results, as our study was within subject and used the same stimuli between conditions. Moreover, our surprisal predictors produced clear N400‐like signatures in the TRFs for the P condition, suggesting that they explained significant variance in the EEG signal related to linguistic processing.

This pop‐out paradigm also came with the disadvantage of including repeated speech segments. For instance, the first two repetitions (NP and C) could have allowed participants to form more precise predictions about what would be heard in the P condition, in turn potentially influencing our second‐stage regression. This was the primary reason for including the sentence repetition when calculating surprisal values for P—to account for the clean speech priors listeners heard in C. Moreover, one may be inherently surprised or allocate more attention to the P condition compared to the NP condition because it is difficult to hear any words in NP. This makes direct interpretation of our surprisal results more complicated than in a standard natural listening experiment (Dou et al. 2025; Gillis et al. 2021; Michaelov et al. 2024; Weissbart et al. 2020). That said, it remains the case that we found significant N400‐like lexical surprisal TRFs and a significant relationship between surprisal and envelope tracking for the P condition but not the NP condition in a within‐subjects design. Thus, despite their likely imperfections, the surprisal values estimated for our pop‐out task still provide strong evidence of linguistic processing and predictive perception in the P condition. Furthermore, even when repetitions were ignored when calculating the surprisal values, we still found that lexical surprisal significantly predicted the EEG activity in our trials and that envelope tracking varied significantly as a function of surprisal. In the end, this finding was not unexpected, given that the surprisal values calculated with and without repetition were moderately and significantly correlated. In sum, regardless of how the surprisal values were derived, lexical surprisal still influenced acoustic processing when given prior information.

It has been the goal of previous studies to find how prior information affects bottom‐up speech processing. To investigate these mechanisms, priors have been manipulated using repetition, attention, or differing the target speech contextually, and target speech has been manipulated by embedding it in noise or by noise vocoding (Cervantes Constantino and Simon 2018; Leonard et al. 2016; Sohoglu et al. 2012; Wang et al. 2019; Wild, Davis, and Johnsrude 2012; Wild, Yusuf, et al. 2012). An advantage of the vocoding paradigm we tested is that the amount of sensory information is kept constant between the conditions of interest (NP and P), allowing us to study the effect of the presence (or absence) of immediate prior information, while controlling for any articulatory variations on the part of the speaker.

Additionally, the modeling framework used in the current study allowed us to directly investigate high‐ and low‐level speech feature processing and how high‐level speech features affected speech encoding when priors were manipulated. Using TRF analyses and a similar vocoding paradigm, Karunathilake et al. (2023) observed stronger late lexical surprisal effects in their primed condition compared to non‐primed (albeit in one hemisphere). This is in line with our present study and supports that higher‐level linguistic representations are engaged when prior information improves intelligibility. In addition, we found evidence of envelope tracking in the EEG but no difference in this tracking between the degraded speech conditions, in line with some previous studies (Di Liberto et al. 2018; Karunathilake et al. 2023; Millman et al. 2015), but counter to others (Baltzell et al. 2017; Corcoran et al. 2023). The present forward modeling‐derived partial correlation approach provided the benefit of increased interpretability with regard to the second‐stage regression results because the effects of surprisal were already removed from the envelope model partial correlations. The second‐stage regression (i.e., the LME model) revealed that, for the primed condition, the envelope tracking of words is stronger for more unpredictable words. This is consistent with the idea that lexical predictions feed back to influence acoustic encoding within the first 100 ms of the word, before higher‐level linguistic processing occurs. Future work using intracranial recordings, for example, might provide additional insight into the nature of these putative top‐down effects especially if it could provide concurrent access to different regions and layers of cortex and, thus, allow for causal interregional analyses. Nevertheless, in line with our initial hypothesis, the second‐stage analysis enabled us to show that the top‐down effects of lexical surprisal on acoustic tracking are not completely explained by within‐speaker variability.

Our findings indicate that the effects of top‐down predictions on bottom‐up sensory encoding cannot be fully explained by articulatory patterns in the speaker. However, the precise mechanisms by which prior knowledge affects sensory processing remain a matter of debate. Specifically, some studies have suggested that prior knowledge sharpens representations in sensory areas (Friston 2005; Kok et al. 2012). Meanwhile, other studies have argued that comparisons between top‐down predictions and bottom‐up input lead to sensory responses in the form of prediction errors (Blank et al. 2018; Blank and Davis 2016; Lupyan and Clark 2015; Rao and Ballard 1999; Sohoglu et al. 2020). Previous research has argued that adjudicating between these two accounts requires varying prior knowledge and speech clarity in a multifactorial manner (Sohoglu et al. 2012). Given that our dataset involved only one level of speech clarity (three‐channel vocoding), we cannot comment on this debate in the present study. Future work using more varied stimuli and/or intracranial recordings will be necessary to explain the mechanisms underlying our primary effect.

Taken together, the present framework may benefit research that seeks to explain symptoms of psychosis such as those seen in schizophrenia. Auditory verbal hallucinations, a common symptom of schizophrenia, are thought to result from an improper weighing of prior information (Corlett et al. 2019; Stephan et al. 2009). Impaired early auditory processing was also found in individuals with schizophrenia, and some believe that these deficits may contribute to the evolution of hallucinations (Javitt and Sweet 2015). The two‐stage regression analysis used here could aid in the evaluation of low‐level speech processing in schizophrenia and demonstrate how prior information is weighed compared to healthy individuals. These methods can also be expanded to investigate predictive processing in other clinical populations such as those with autism spectrum disorder.

## Author Contributions

**Michael P. Broderick:** formal analysis, validation, writing – review and editing, writing – original draft. **Edmund C. Lalor:** validation, writing – review and editing, project administration. **Shyanthony R. Synigal:** writing – original draft, writing – review and editing, formal analysis, validation.

## Funding

This work was supported by Del Monte Institute for Neuroscience, Government of Ireland Postgraduate scholarships from the Irish Research Council, and Simons Foundation Autism Research Initiative.

## Conflicts of Interest

The authors declare no conflicts of interest.

## Supporting information


**Figure S1:** Additional LME model coefficients. This figure displays other fixed effect coefficients from the LME model in both the 0–100 ms and 100–200 ms time ranges. These include envelope variability (envStd), relative pitch (f_rel_), resolvability (res), the interaction between surprisal and envelope variability (surp*envStd), and the interaction between relative pitch and resolvability (f_rel_*res). The white markers indicate channels deemed significant by the LME model, and the black markers indicate channels that survived cluster‐based permutation tests.

**Figure S2:** LME model surprisal coefficients for the NP and P conditions. In this case, the NP lexical surprisal values were also used in the P condition. This treats it as if there was no repetition effect of the audio when calculating the lexical surprisal values, unlike what was done in Figure 3B. The black markers indicate channels that survived cluster‐based permutation tests.

## Data Availability

The data and scripts associated with this study are available at https://doi.org/10.17605/OSF.IO/756XN.
