# Supplementary figures and images for: Quantifying the Influence of Lexical Surprisal on Acoustic Speech Encoding While Controlling for Within‐Speaker Variability
Source: Eur J Neurosci. 2026 Jul 2;64(1):e70569. doi: 10.1111/ejn.70569 (PMC13325525; doi:10.1111/ejn.70569)

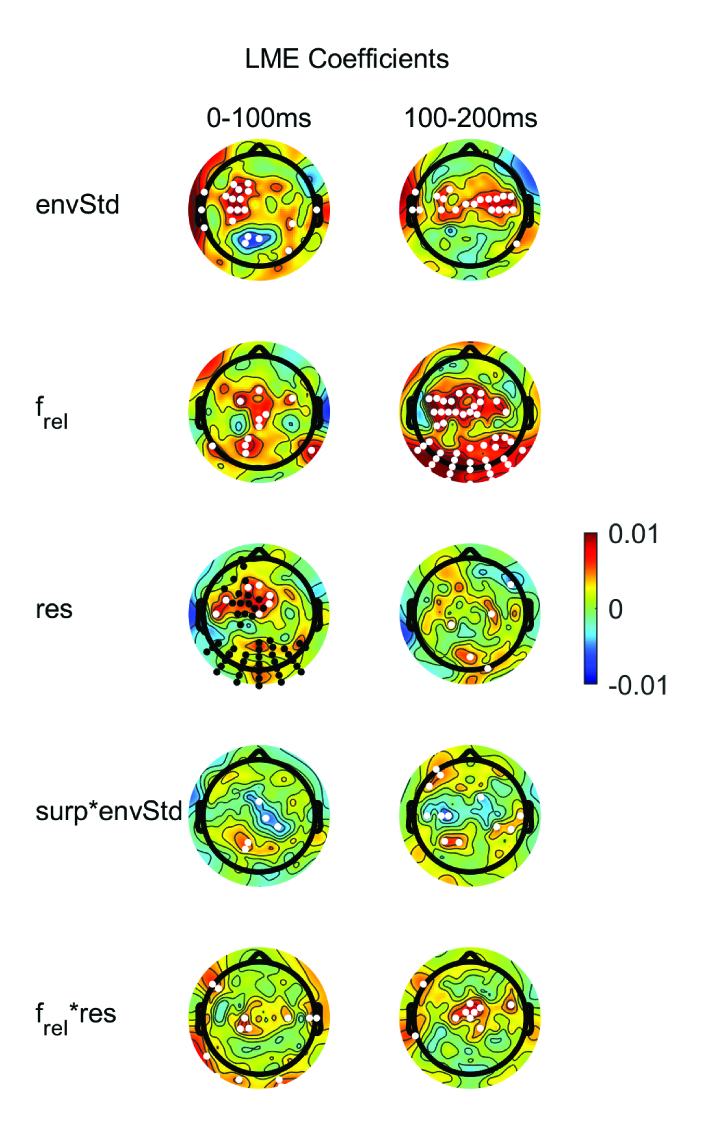

Supplement: Supplementary file 1 — Figure S1: Additional LME model coefficients. This figure displays other fixed effect coefficients from the LME model in both the 0–100 ms and 100–200 ms time ranges. These include envelope variability (envStd), relative pitch (frel), resolvability (res), the interaction between surprisal and envelope variability (surp*envStd), and the interaction between relative pitch and resolvability (frel*res). The white markers indicate channels deemed significant by the LME model, and the black markers indicate channels that survived cluster‐based permutation tests. [file EJN-64-0-s002.tif]

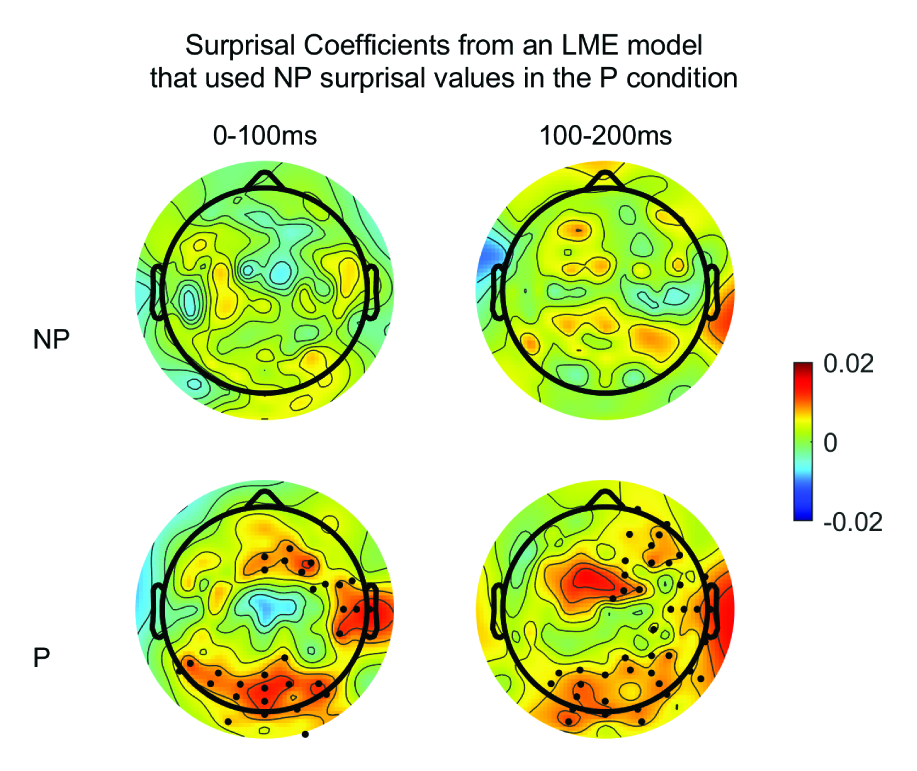

Supplement: Supplementary file 2 — Figure S2: LME model surprisal coefficients for the NP and P conditions. In this case, the NP lexical surprisal values were also used in the P condition. This treats it as if there was no repetition effect of the audio when calculating the lexical surprisal values, unlike what was done in Figure 3B. The black markers indicate channels that survived cluster‐based permutation tests. [file EJN-64-0-s001.tif]
